# Dusp8 affects hippocampal size and behavior in mice and humans

**DOI:** 10.1038/s41598-019-55527-7

**Published:** 2019-12-20

**Authors:** Peter Baumann, Sonja C. Schriever, Stephanie Kullmann, Annemarie Zimprich, Annette Feuchtinger, Oana Amarie, Andreas Peter, Axel Walch, Valerie Gailus-Durner, Helmut Fuchs, Martin Hrabě de Angelis, Wolfgang Wurst, Matthias H. Tschöp, Martin Heni, Sabine M. Hölter, Paul T. Pfluger

**Affiliations:** 1 Research Unit Neurobiology of Diabetes, Helmholtz Zentrum München, 85764 Neuherberg, Germany; 2 Institute for Diabetes and Obesity, Helmholtz Zentrum München, 85764 Neuherberg, Germany; 3 German Center for Diabetes Research (DZD), 85764 Neuherberg, Germany; 4 Neurobiology of Diabetes, TUM School of Medicine, Technische Universität München, 80333 Munich, Germany; 5 Institute for Diabetes Research and Metabolic Diseases (IDM) of the Helmholtz Center Munich at the University of Tübingen, 72076 Tübingen, Germany; 6 Department of Internal Medicine IV, University Hospital of Tübingen, 72076 Tübingen, Germany; 7 German Mouse Clinic, Institute of Experimental Genetics, Helmholtz Zentrum München, 85764 Neuherberg, Germany; 8 Institute of Developmental Genetics, Helmholtz Zentrum München, 85764 Neuherberg, Germany; 9 Chair of Developmental Genetics, Technische Universität München-Weihenstephan, c/o Helmholtz Zentrum München, 85764 Neuherberg, Germany; 10 Research Unit Analytical Pathology, Helmholtz Zentrum München, 85764 Neuherberg, Germany; 11 Institute for Clinical Chemistry and Pathobiochemistry, Department for Diagnostic Laboratory Medicine, University Hospital of Tübingen, 72076 Tübingen, Germany; 12 Chair of Experimental Genetics, School of Life Science Weihenstephan, Technische Universität München, 85354 Freising, Germany; 13 German Center for Neurodegenerative Diseases (DZNE) Site Munich, 81377 Munich, Germany; 14 Munich Cluster for Systems Neurology (SyNergy), Ludwig-Maximilians-Universität München, 81377 Munich, Germany; 15 Division of Metabolic Diseases, Technische Universität München, 80333 Munich, Germany

**Keywords:** Cognitive control, Hippocampus

## Abstract

Dual-specificity phosphatase 8 (Dusp8) acts as physiological inhibitor for the MAPKs Jnk, Erk and p38 which are involved in regulating multiple CNS processes. While Dusp8 expression levels are high in limbic areas such as the hippocampus, the functional role of Dusp8 in hippocampus morphology, MAPK-signaling, neurogenesis and apoptosis as well as in behavior are still unclear. It is of particular interest whether human carriers of a *DUSP8* allelic variant show similar hippocampal alterations to mice. Addressing these questions using Dusp8 WT and KO mouse littermates, we found that KOs suffered from mildly impaired spatial learning, increased locomotor activity and elevated anxiety. Cell proliferation, apoptosis and p38 and Jnk phosphorylation were unaffected, but phospho-Erk levels were higher in hippocampi of the KOs. Consistent with a decreased hippocampus size in Dusp8 KO mice, we found reduced volumes of the hippocampal subregions subiculum and CA4 in humans carrying the *DUSP8* allelic variant SNP rs2334499:C > T. Overall, aberrations in morphology and behavior in Dusp8 KO mice and a decrease in hippocampal volume of SNP rs2334499:C > T carriers point to a novel, translationally relevant role of Dusp8 in hippocampus function that warrants further studies on the role of Dusp8 within the limbic network.

## Introduction

Dual-specificity phosphatases (Dusps) are a heterogeneous group of protein phosphatases that dephosphorylate phospho-tyrosine as well as phospho-threonine/phospho-serine residues within a single protein. Dusps play major roles in cellular signaling and can be divided into six subgroups: slingshot phosphatases, phosphatase of regenerating liver (PRL), Cdc14 phosphatases, phosphatase and tensin homologue deleted on chromosome 10 (PTEN)-like and myotubularin phosphatases, atypical Dusps, and mitogen-activated protein kinase phosphatases (MKP)^1^. The MKP family consists of at least ten Dusps that show a diverse pattern of tissue expression and target specificity. MKPs are classified as potent inhibitors of mitogen-activated protein kinases (MAPK) such as p38, extracellular-signal regulated kinase (ERK) or c-jun-N-terminal kinase (Jnk) which are involved in cellular processes ranging from proliferation, differentiation, survival and apoptosis to inflammatory responses^2,3^. MAPK are considered as major regulators of the cellular stress response and have been linked with numerous inflammatory and metabolic disorders ranging from cancer to neurodegenerative diseases^3–8^.

In this study, we aimed to interrogate the physiological role of the MKP family member Dusp8 on the CNS control of behavior. Our interest in Dusp8 was driven by its target specificity towards MAPKs, by its predominant expression in the central nervous system^9^, and by the relative paucity of available literature on the function of Dusp8 within the CNS. To specifically address whether Dusp8 plays a role in central MAPK signaling and in the control of behavior, we used a global knockout (KO) mouse model of Dusp8. Prompted by the high expression of Dusp8 in the limbic system and especially the hippocampus, we focused our studies on evaluating the impact of Dusp8 ablation on cognitive functions, i.e. spatial and reverse task learning and memory performance, and on anxiety behavior. We further wanted to address whether Dusp8 deficiency is having an impact on neuronal activity and neurogenesis in the hippocampus. Last, we aimed to assess whether Dusp8 ablation in mice leads to general morphological changes in the hippocampus, and whether such changes in hippocampus morphology can also be found in human carriers of an allelic variant of DUSP8^10^.

## Results

### Increased locomotion and impaired spatial learning in female Dusp8 KO mice

We first aimed to assess whether Dusp8 deficiency can perturb behaviors in mice that are typically assigned with the limbic system. Our efforts concentrated on learning and memory behaviors in a social, group-housed environment^11^. The testing was conducted within a TSE IntelliCage system for automated behavioral monitoring in the home cage of unstressed group-housed mice. Because of inter-male aggression and the animal ethics guidelines of our institution, our studies in the IntelliCage setup were limited to female mice. However, prior to the IntelliCage tests we carried out a social discrimination task in both male and female mice (Suppl. Fig. 1A). The exposure to a single ovariectomized female stimulus mouse in a neutral cage for 4 minutes revealed comparable trends towards a reduced interaction time with the unfamiliar mouse in both male and female Dusp8 KO mice in the sample phase (Suppl. Fig. 1B–E). Notably, stimulation with a second, familiar mouse did not yield the expected decrease in interaction time in both wild type (WT) and KO mice. Overall, we observed comparable calculated discrimination indices in Dusp8 KO and Dusp8 WT mice (Suppl. Fig. 1F,G) in both sexes.

**Figure 1:**
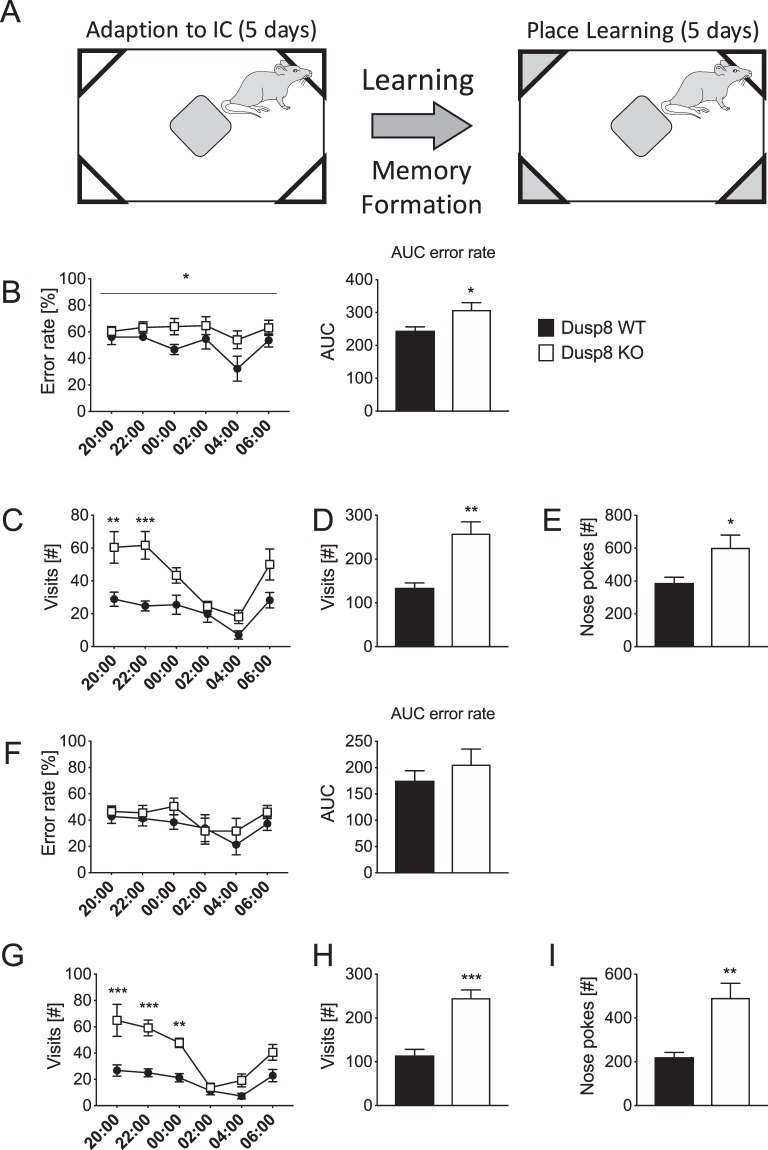
Place Learning Test of female Dusp8 WT and KO mice group-housed in the automated IntelliCage behavioral monitoring system. (**A**) Schematic of the place learning task. White corners contain water bottles freely accessible after a nose poke during the adaption phase (first 5 days). In the subsequent place learning task phase (duration 5 days), corners with blocked access to the water bottles after a nose poke are shown in grey. (**B**) Percentage of erroneous nose pokes in the first night phase as measure for the learning performance. Overall activity of the mice in the first night phase is monitored as bi-hourly number of corner visits (**C**), average number of corner visits (**D**) and average number of nose pokes (**E**). (**F**–**I**) depict the corresponding values from the second night of the place learning task. WT: n = 8, KO: n = 9. Means ± SEM. *p < 0.05; **p < 0.01; ***p < 0.001.

Testing for spatial learning and memory (Fig. 1A) in the IntelliCage set up revealed a significantly increased error rate for Dusp8 KO mice compared to WT control animals in the first night of the place learning task (Fig. 1B), pointing towards an impairment in spatial memory. Next to the differences in memory formation, we saw differences in the overall activity between female Dusp8 WT and KO mice. Significantly higher numbers of visits of all test corners and increased numbers of nose pokes for accessing the drinking bottles reflect a higher locomotor activity in Dusp8 KO mice (Fig. 1C–E). This increase in activity was present only in the first hours of the dark phase. After that, the number of nose pokes and visits dropped down to the level of WT animals. The absence of differences in error rate in the second night (Fig. 1F) may indicate that the retrieval of stored memory is still intact in Dusp8 KO mice. The different activity pattern of the first night phase was also present in the second night (Fig. 1G–I) and persisted over the entire period of testing in the IntelliCage (data not shown). These results suggest an impaired spatial memory formation and increased locomotor activity in female Dusp8 KO mice.

Five days after the place learning task, female Dusp8 WT and KO mice were challenged with a reversal learning task by shifting access to the drinking bottles in the IntelliCage system to the opposite corner of the cage of the previously correct one (Suppl. Fig. 2A). Dusp8 KO mice did not show any alterations in the error rates in the first night of the reversal learning task (Suppl. Fig. 2B). However, Dusp8 KO females showed a significantly higher number of initial visits to the test corners at the onset of the night phase. In the later period of the night, after eight hours of night phase, the number of visits by the Dusp8 KO mice was equal to the number of visits by WT control mice (Suppl. Fig. 2C). Overall activity was higher in Dusp8 KO mice compared to WT control animals (Suppl. Fig. 2D,E). Error rates in the second night did not differ between genotypes (Suppl. Fig. 2F). The persistent high activity rate in the second night (Suppl. Fig. 2G–I) is in agreement with the observations from the preceding place learning task. These results suggest an intact cognitive flexibility, as Dusp8 WT and KO mice can readily adapt the already established memory to the new reversal task. Taken together, the data indicate that Dusp8 has no influence on memory flexibility but contributes to an increased locomotor drive in female Dusp8 KO mice.

### Male and female Dusp8 KO mice have an unperturbed spatial orientation in the Y-maze

**Figure 2:**
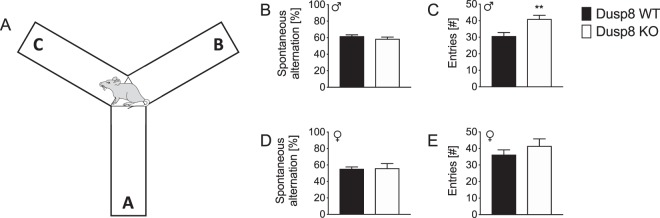
Spatial working memory of male and female Dusp8 WT and KO mice in a Y-Maze. (**A**) Mice were allowed to freely explore the Y-Maze arena during the test phase. Arms were assigned with letters A to C to record the respective arm entries. (**B**–**E**) Orientation and horizontal investigation performance in the arena, measured as percentage of spontaneous alternations between arms in male (**B**) and female (**D**) mice, and as total number of arm entries in male (**C**) and female (**E**) mice. Male WT: n = 15, male KO: n = 12; female WT: n = 8, female KO: n = 12. Means ± SEM. **p < 0.01.

The mild impairment of spatial memory formation in female Dusp8 KO mice in the home cage set up prompted us to next assess whether Dusp8 deficiency affects active spatial exploration, retrograde working memory and anxiety using test protocols for individually tested mice, which allowed the inclusion of male mice in the study. We conducted a spontaneous alternation test using a Y-maze test (Fig. 2A) in male and female Dusp8 WT and KO mice. In this spatial memory task, we found the same percentage of spontaneous arm alternations of Dusp8 KO mice compared to WT controls in both sexes (Fig. 2B,D). Nonetheless, male, but not female Dusp8 KO mice showed a significantly higher number of arm entries (Fig. 2C,E). Overall, these data show intact spatial orientation and higher locomotion in Dusp8 KO mice, and suggest increased exploratory behavior of novel environments.

### Dusp8 KO mice have an intact short- and long-term memory in the object recognition test

**Figure 3:**
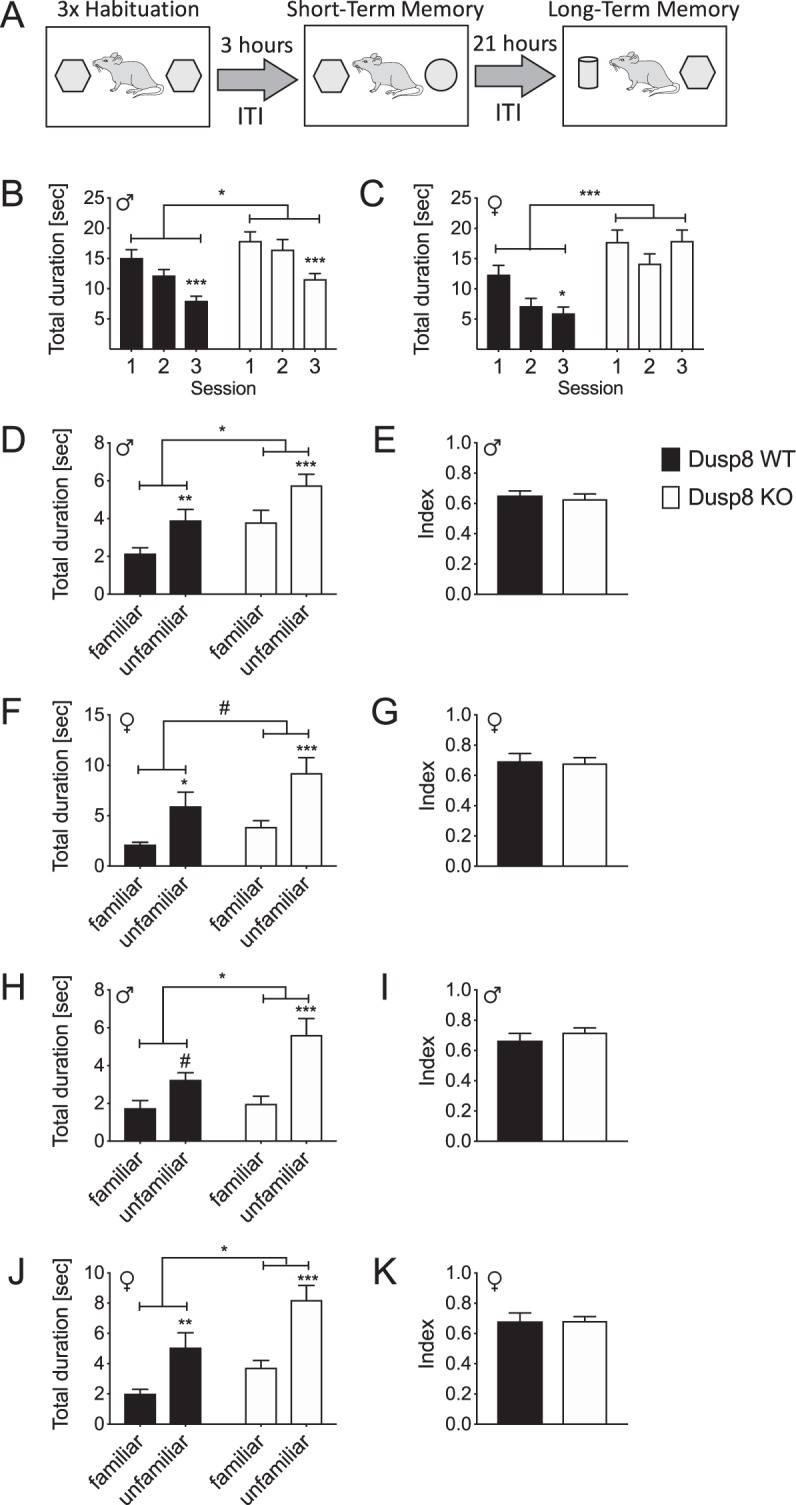
Object Recognition Test in female and male Dusp8 WT and KO mice. (**A**) Protocol of the object recognition task with three habituation sessions 1–3 followed by a short and a long-term memory test after a 3-hour or 24-hour inter-trial interval (ITI), respectively. (**B**,**C**) Interaction times with identical objects in the testing arena for the habituation sessions 1–3 in male (**B**) and female (**C**) mice, respectively. (**D**–**G**) Short-term memory recognition measures after the 3h-ITI for the familiar and a novel object, shown as total duration spent with the familiar and unfamiliar object, or as object recognition index in male (**D**,**E**) or female (**F**,**G**) mice. (**H**–**K**) depicts the corresponding long-term memory recognition measures after the 24h-ITI with the familiar and a novel unfamiliar object. Male WT: n = 14, male KO: n = 15; female WT: n = 8, female KO: n = 11. Means ± SEM. # p < 0.1; *p < 0.05; **p < 0.01; ***p < 0.001.

Prompted by the higher exploratory behavior in the Y-maze, we next aimed to assess the short- and long-term working memory and exploratory behavior of female and male Dusp8 WT and KO mice exposed to inanimate objects in an object recognition test (Fig. 3A). In the habituation sessions 1 to 3, male and female WT control mice familiarized with the objects and thus decreased their time of investigation with each session (Fig. 3B,C). Male Dusp8 KO mice also decreased their investigation time per habituation session but showed an overall longer investigation time of the two presented identical inanimate objects relative to their WT controls (Fig. 3B). In contrast, female Dusp8 KO mice did not decrease their time of investigation during the three habituation sessions compared to WT controls (Fig. 3C).

When presented with a new unfamiliar object three hours after the last habituation session, male and female Dusp8 KO mice showed higher interaction times compared to their WT counterparts (Fig. 3D,F), but comparable indices for object recognition (Fig. 3E,G). These data suggest higher locomotor activity and an intact short-term memory. We found similar results for long-term memory tested 24 hours after the last habituation session, i.e. comparable indices of object recognition (Fig. 3I,K), but higher interaction times in male and female Dusp8 KO compared to WT mice (Fig. 3H,J). Taken together, the object recognition task revealed an intact object recognition short- and long-term memory in male and female Dusp8 KO mice for inanimate objects and an overall higher locomotor activity compared to Dusp8 WT controls.

### Male Dusp8 KO mice show increased anxiety in the open field test

**Figure 4:**
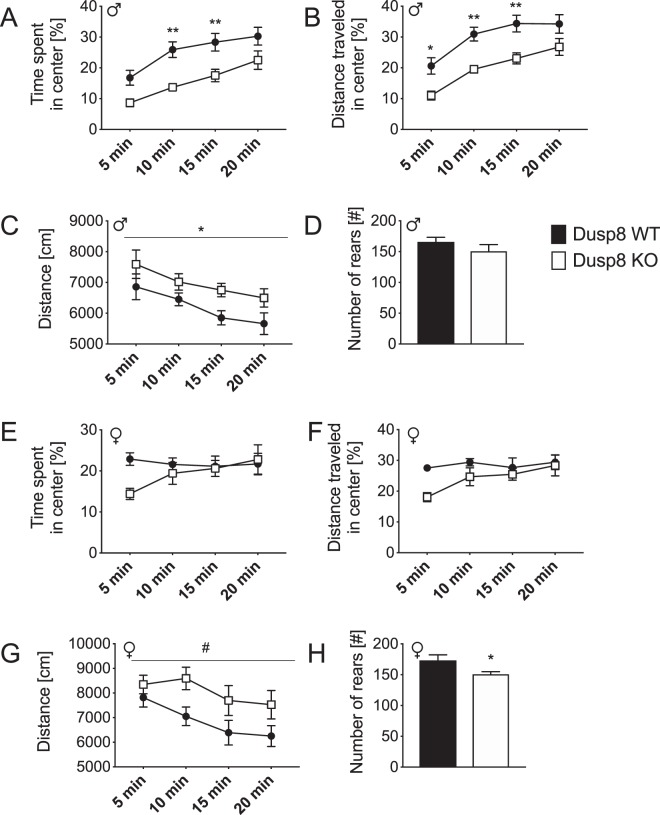
Open Field Test in female and male Dusp8 WT and KO mice. Percentage of time spent (**A**,**E**) and percentage of distance travelled (**B**,**F**) in the center of the open field during the test time of 20 minutes in male and female Dusp8 WT and KO mice. Total activity of male (**C**,**D**) and female (**G,H**) Dusp8 WT and KO mice is shown as distance travelled in the entire arena during the test time (**C**,**G**) and the total number of rearing events (**D**,**H**). Male WT: n = 15, male KO: n = 14; female WT: n = 8, female KO: n = 12. Means ± SEM. ^#^p < 0.1; ^*^p < 0.05; ^**^p < 0.01.

We next aimed to address in an Open Field Test, whether Dusp8 deficiency can affect anxiety behavior in a novel environment. Male Dusp8 KO mice spent significantly less time in the anxiogenic, exposed center of the arena and traveled a significantly lower distance in the center than WT controls (Fig. 4A,B). The male Dusp8 KO mice further displayed a significantly higher total distance traveled in the arena (Fig. 4C) but no difference in rearings (Fig. 4D) compared to male WT controls. In contrast, female Dusp8 KO mice showed no difference in time spent in the center of the arena and the distance traveled in the center (Fig. 4E,F). Comparable to their male WT littermates, female Dusp8 KO mice tended to move around more in the arena (Fig. 4G). Contrary, the number of rearing events was lower in female Dusp8 KO compared to their WT controls (Fig. 4H). Overall, our data indicate increased anxiety levels and locomotor activity in male Dusp8 deficient mice that are independent from a social context whereas Dusp8 female mice show a tendency to an increased locomotor activity but decreased vertical investigation.

### Mildly impaired vision but comparable startle response and prepulse inhibition in Dusp8 KO mice

Prompted by our finding of mildly increased anxiety in Dusp8 KO mice, we next investigated the sensorimotor gating of Dusp8 KO mice by testing their startle response to an acoustic stimulus. We further challenged the mice with a prepulse stimulus and assessed the level of prepulse inhibition of the startling stimulus. Both male and female Dusp8 WT and KO mice showed comparable startle responses (Suppl. Fig. 3A,C) and prepulse inhibition (Suppl. Fig. 3B,D), which indicates unperturbed sensorimotor gating in mice lacking Dusp8. In addition to sensorimotor gating, we further tested the vision of the mice to exclude a bias due to missing eyesight. A virtual drum test revealed a significantly reduced performance in male and female Dusp8 KO and Dusp8 WT mice (Suppl. Fig. 3E,F). However, animals were still able to perform the test and achieved visual acuity indicates a remaining eyesight of Dup8 KO mice. These results show reaction impairment in the visual test as readout for reduced vision.

### Decreased hippocampus weight and volume in global Dusp8 KO mice

**Figure 5:**
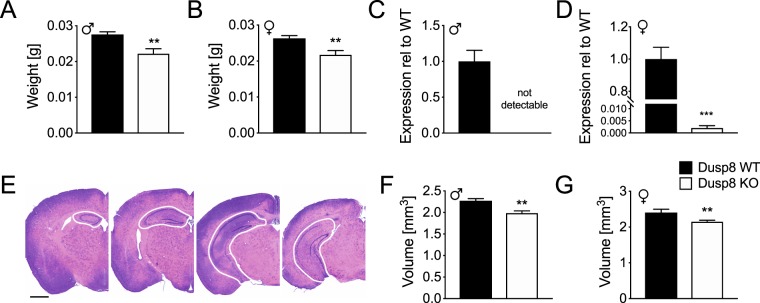
Hippocampus weights and volumes in male and female Dusp8 WT and KO mice. Tissue weights were recorded from freshly microdissected hippocampi (**A**,**B**). Male WT: n = 7, male KO: n = 6; female WT: n = 7, female KO: n = 7. The knockout of Dusp8 was confirmed in hippocampus lysates using qPCR after RNA extraction. (**C**,**D**) H&E stainings of sequential cryosections through the brain (**E**, scale bar 1 mm) were used to calculate hippocampal volumes of male (**F**) and female (**G**) Dusp8 KO and Dusp8 WT mice. Male WT: n = 5, male KO: n = 7, female WT: n = 4, female KO: n = 6. Means ± SEM. **p < 0.01, ***p < 0.001.

Prompted by behavioral changes that are often associated with the limbic system and hippocampal function, we next investigated the size of the hippocampus using microdissection of fresh brain tissues of chow-fed male (WT: 34.4 ± 1.3 g; KO: 35.3 ± 1.5 g) and female Dusp8 WT and KO mice (WT: 27.5 ± 1.3 g; KO: 27.9 ± 1.1 g). Weighing the dissected brain tissue revealed reduced hippocampal weights in male and female Dusp8 KO mice, compared to WT littermates (Fig. 5A,B). The differences in hippocampal weights between genotypes remained significant after controlling for body weight and skull length (Males F(1,11) = 20.564, p < 0.001; females F(1,12) = 5.215, p = 0.041). We confirmed the knockout of Dusp8 in lysates of microdissected hippocampi by qPCR in male (Fig. 5C) and female (Fig. 5D) Dusp8 KO mice. Additionally, we assessed sequential H&E-stained hippocampal sections (Fig. 5E), and found smaller hippocampal volumes in male and female Dusp8 KO compared to WT mice (Fig. 5F,G). Again, differences in hippocampal volumes remained significant between Dusp8 WT and KO mice after controlling for BW (Males F(1,9) = 12.125, p = 0.007; females F(1,7) = 27.911, p < 0.001).

Protein extracts from total dissected hippocampi were subsequently analyzed for phosphorylated and un-phosphorylated MAPKs or the housekeeper GAPDH by western blotting, and revealed unperturbed Jnk- (Suppl. Fig. 4A,B) and p38-signaling (Suppl. Fig. 4C,D) in hippocampi of male and female Dusp8 KO mice. Notably, we found a tendency to higher phosphorylation of Erk in hippocampal lysates of male Dusp8 KO mice (p = 0.081; Suppl. Fig. 4E) and a significantly increased phosphorylation in female Dusp8 KO mice compared to Dusp8 WT animals (p = 0.0028; Suppl. Fig. 4F). Immunohistochemical staining of cryosections for Ki67 (Suppl. Fig. 5A), a marker for cell mitosis, revealed similar proliferation rates in the dorsal and ventral hippocampus of male (Suppl. Fig. 5B,C) and female (Suppl. Fig. 5D,E) Dusp8 WT and KO mice, respectively. Immunohistological staining for apoptosis marker caspase 3 did not show any positive cells in hippocampi of male and female Dusp8 WT and KO mice (data not shown). These observations suggest comparable Jnk and p38 activity as well as cell proliferation and cell death rates in hippocampi of male Dusp8 WT and KO mice. Whether chronically activated Erk-signaling in hippocampi of Dusp8 KO mice plays a causal role in decreasing hippocampus morphology remains to be determined.

Adult Dusp8 KO and Dusp8 WT mice had comparable body weights throughout the study (Suppl. Fig. 6A). Young 21-days-old Dusp8 KO mice had reduced hippocampus weights (Suppl. Fig. 6B) and body weights (Suppl. Fig. 6C) compared to their WT littermates. However, after normalization of the hippocampus weight to the body weight Dusp8 WT and KO mice displayed a comparable hippocampus mass (Suppl. Fig. 6D). Overall, our finding of a reduced normalized hippocampus size in adult but not young Dusp8 KO mice points to a maturation-caused effect rather than an impairment in hippocampus development.

### Association of genetic variation in the *DUSP8* locus with hippocampal volume in humans

**Figure 6:**
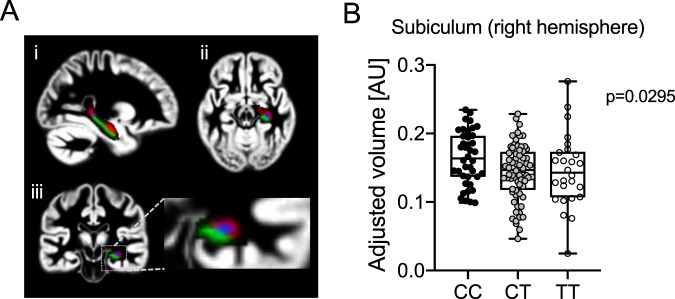
Association of hippocampal volume and *DUSP8* SNP rs2334499:C > T in humans. (**A**) Normalized high-resolution T1 brain scan in (i) sagittal, (ii) horizontal, and (iii) coronal view (color code: red = CA1–3, blue = CA4, green = subiculum). (**B**) Box-Whisker plots (Median, max/min) of hippocampal volumes of patients carrying SNP rs2334499:C>T adjusted to grey matter volume, sex, age, and BMI (CC: n = 40, CT: n = 68, TT: n = 26).

To translate our mouse data to the human situation, we next used high-resolution T1 images of brain scans to analyze the hippocampal volume of human volunteers (Fig. 6A). Participants of this TUEF cohort at the University Hospital Tübingen were genotyped for the single nucleotide polymorphism (SNP) rs2334499:C > T that is located upstream of the *DUSP8* gene, and revealed a frequency of 0.3 vs. 0.2 for the C vs. T allele. Subsequently, after adjusting for whole brain gray matter volume, we identified an association between SNP rs2334499:C > T and the volume of the hippocampal subregion subiculum (Fig. 6B, right hemisphere p = 0.0185; left hemisphere p = 0.09). This association remained statistically significant after additional adjustments for age, sex, and BMI (right hemisphere p = 0.0295; left hemisphere p = 0.09). A further statistically significant association was detected for subregion CA4-dentate gyrus (left hemisphere p = 0.0389) that remained significant after additional adjustments for age, sex, and BMI (p = 0.0388). No significant association was found for the subregions CA1-3 (data not shown). Overall, this data suggests that *DUSP8* SNP rs2334499:C > T is linked to a reduction of the volume of hippocampal subregions in humans.

## Discussion

In this study we characterized the influence of Dusp8, a physiological inhibitor of MAPK signaling that is mainly expressed in the brain^12^, on cognitive performance and anxiety behavior in mice. Our behavioral assessment of Dusp8 WT and KO mice revealed mildly impaired spatial memory formation and visual performance, consistently higher locomotor activity in a variety of tests, and increased anxiety in Dusp8 deficient mice. These behavioral alterations were associated with a reduced hippocampus mass and volume in Dusp8 KO compared to WT controls. Human minor allele carriers of the *DUSP8* SNP rs2334499:C > T had reduced volumes of the hippocampal subregions subiculum and CA4. In contrast to reports on a close bidirectional modulation of Dusp8 and Jnk^1,2^, we found comparable levels of p38 and Jnk phosphorylation in hippocampi of Dusp8 WT and KO mice, but increased levels of phospho-Erk.

The hippocampus is a major area involved in cognitive behaviors and anxiety control. In our Dusp8 deficient mouse model, we found a reduction in the size of the hippocampus which is known to govern emotional output, social interaction and anxiety^13,14^. Hippocampal dysfunction has been associated with anxiety as well as mood and cognitive disorders in humans^15,16^. We found higher anxiety levels in male Dusp8 KO mice and higher locomotor activity in male and female Dusp8 KO mice, which resonates with a previous report that showed elevated spontaneous behavior patterns in mice subjected to surgical hippocampus lesions^17^. The latter report^17^ further revealed impaired aversive learning as well as spatial learning in mice with hippocampal lesions, which is partially consistent with modest impairments in spatial learning in our Dusp8 KO model. In the past, hippocampal lesions were also associated with an impaired performance in spatial memory tests that require visual cues^18^. Notably, we also found an impaired performance in a visual drum test in Dusp8 KO mice. Such data could point towards impaired eyesight in our KO mice. Hippocampal circuits are however also known to control visual discrimination in a paradigm built upon complex spatial stimuli^19^. Accordingly, Dusp8 deficiency may impair hippocampal processes governing visual recognition without directly affecting eyesight. Future studies should interrogate the role of Dusp8 as regulator of visual performance and eyesight, and should further assess whether these effects are interlinked with deficits in cognitive behaviors and anxiety control. Overall, our findings are largely consistent with murine hippocampal lesion models, suggesting that Dusp8 plays a regulatory role in hippocampal neurocircuitry governing social, spatio-visual and cognitive behaviors. Notably, however, neurocircuits in the hippocampus of Dusp8 KO mice appear largely intact, as evidenced by our finding of unaffected apoptosis and cell proliferation.

Male Dusp8 KO mice show more pronounced anxiety relative to their WT littermates compared to female mice, where we see only a trend for anxiety in KO compared to WT mice. These results indicate an influence of sex, but the differences in anxiety behavior between male and female KOs are modest, and may rather be explained by differences in housing and age^20,21^. Almost half of the male mice were kept in single-housed conditions due to inter-male aggression, while female animals were always group-housed. Moreover, female mice were three to four months younger than the tested males. Overall, the roles of sex and age for the behavioral aberrations in Dusp8 KO mice remain elusive. Only modest differences in phenotypes in the female and male Dusp8 KO mice nonetheless make it more likely that common mechanistic underpinnings exist that drive anxiety behavior and locomotion in male and female Dusp8 KO mice.

Dusp8 is a physiological inhibitor of MAPK-signaling. In hippocampus lysates, we found comparable phosphorylation levels of p-Jnk and p-p38, but elevated Erk phosphorylation levels in Dusp8 KO mice compared to Dusp8 WT mice. Our data is thus in contrast to earlier reports that show specificity of Dusp8 towards p38 and Jnk^3,22,23^. However, our data agrees with a recent report^24^ that revealed impaired Erk phosphorylation in the cardiac muscle of Dusp8 KO mice and a major Erk-gated role for Dusp8 in the ventricular remodeling in the heart. Overall, these data indicate context- and tissue-specific *in vivo* activity of Dusp8 towards Erk, at least for the murine heart and hippocampus. However, increased Erk-signaling in Dusp8 KO does not appear to influence proliferation and apoptosis rates of the hippocampus, as evidenced by comparable levels of neuronal proliferation marker Ki67 and a complete lack of apoptosis marker Casp3. The question of why Dusp8 KO mice have a reduced hippocampus volume is thus still unclear, and requires further investigation. Such studies should further reveal whether Dusp8 differentially affects MAPK-signaling in the different hippocampal cell layers and cell types.

Taken together, we found increased anxiety and hyperactivity as well as mildly, but significantly decreased spatial memory performance, visual performance and decreased hippocampal volume and mass in mice deficient for Dusp8. The observed behavioral and morphological aberrations in mice were independent from neurogenesis or apoptosis. Notably, we were further able to show that hippocampal volumes for the subiculum and CA4 in human subjects were associated with the minor allele variant of *DUSP8* (SNP rs2334499:C > T). SNP rs2334499:C > T is located upstream to the *DUSP8* gene on human chromosome (GRCh38:CM000673.2; Chromosome 11: 1,554,051–1,572,271 reverse strand). However, since *DUSP8* is positioned on the negative strand, SNP rs2334499:C > T has been allocated to this gene^25^. Whether this allocation is also of functional relevance in terms of altered gene expression is nonetheless still unclear. Quantitative PCR analyses of *DUSP8* in human hippocampus specimens of CC, CT and TT allele carriers are thus warranted. Such post-mortem studies could be complemented with cell culture studies in appropriate hippocampal neuronal cell lines with genetic modulation of SNP rs2334499, or in human inducible pluripotent stem cells (hiPSCs) derived from skin biopsies that are transdifferentiated into hippocampal neurons representative for the CC, CT or TT alleles. Further, whether the observed reduction in hippocampal size has any influence on behavior and cognition in humans still has to be evaluated. To date, there is no data available connecting SNP rs2334499:C > T and alterations in behaviors governed by the hippocampus, and our MRI cohort was too small to assess behavioral alterations in association with *DUSP8* variants. Our current findings nonetheless provide guidance for future studies in larger numbers of subjects which can be directed towards deciphering the complex association between genetic risk-variants, brain morphology and potential behavioral traits.

## Methods

### Animals

Dusp8 global WT and KO mice were generated as described^24^. All mice in our studies were littermates derived from heterozygous parents on a C57BL/6 J background. Mice were bred and group-housed on a 12:12 h light-dark cycle (dark phase from 6 am to 6 pm) at 22 °C and 50% to 60% air humidity with free access to chow diet (Altromin, #1314) and water. Male mice were partially single-housed due to inter-male aggression (13 out of 29 mice). All samples for protein measurements, weight and volume calculation were taken from the colony of mice tested for behavior after completing the tests. Cohorts were weight matched and the age of female and male mice ranked from 9 to 11 months and females tested in IntelliCage from 3 to 5 months at the start time of testing. The second cohort of mice tested in the virtual drum test was 6–7 months old at the time of testing. The murine studies were based on power analyses to assure adequate sample sizes, performed in accordance with relevant guidelines and regulations, and approved by the Animal Ethics Committee of the Government of Upper Bavaria, Germany (animal protocol number TVA 55.2-1-54-2532-46-16 and VTA 55.2–2532.Vet_02-16-80).

### Assessing cognition in mice

#### Open field test

Open field test was performed between 08.00 am–02.00 pm. Mice were placed in the open field arena at the middle position of the hind wall (facing the wall). The locomotor activity of the mouse was automatically tracked for 20 min by using a light beam break ActiMot Box System (TSE ActiMot Version 08.00, TSE Systems, Bad Homburg, Germany) as previously described^26^. Prior to testing, light conditions were set to 200 lx in the middle of the arena.

#### Y-maze

Spontaneous alternations in the Y-maze were tested and analyzed according to Holter *et al*.^27^ and conducted between 08.00 am–02.00 pm. Tested mice were introduced in a Y-shaped maze facing the wall of the starting arm. Arm entries were manually recorded for 5 min. Starting arms were alternated.

#### Object recognition memory test

Object recognition memory test was performed between 08.00 am-02.00 pm. Two identical objects were presented three times to the test mouse in a neutral test cage for 5 min with an inter-trial interval of 15 min. The first test phase started 3 h after the last sample session. An unfamiliar object was presented together with one familiar object for 5 min. The second test phase was performed 24 h after the last sample session. Another unfamiliar object was presented together with the familiar object. Interaction times with the different objects were recorded manually with a handheld computer PSION Teklogix Workabout Pro (Noldus Pocket Observer Version 2.1.23.f). Tests were performed according to a previously published protocol by Holter and colleagues^27^.

#### IntelliCage habituation

Tests for spatial learning in the IntelliCage system (IntelliCagePlus Version 3.3.2.0, TSE Systems, Bad Homburg, Germany) were performed with female mice only. Mice were group-housed up to 10 animals per cage in genotype-mixed groups. 3 d prior to the habituation in the IntelliCage, all mice were subcutaneously implanted with passive transponders that allowed distinguishing mice entering the four IntelliCage corners. Before starting the test programs, mice were allowed to habituate for 3 d in the IntelliCage with free access to food and water. Following habituation, mice were trained to get access to drinking spouts in all four corners after a nose poke action.

#### Place learning tasks

Place Learning was conducted by assigning each mouse to one corner of the cage for access to the drinking bottles after a nose poke. Assignments were equally distributed over the cage, and nose pokes and corner visits were automatically tracked and recorded by the IntelliCage software for 5 d. Mice were not disturbed or handled during testing days. Tests were performed according to a previously published protocol by Holter and colleagues^27^.

### Hippocampus volume calculation

Brains were harvested 10 days after the end of the last test session. Mice were euthanized in CO_2_ and perfused with 4% paraformaldehyde (PFA) after a saline wash. Brains were removed and post-fixed overnight in 4% PFA at 4 °C. After equilibration with 30% sucrose, brains were cut coronally in a cryostat into 20 μm sections. Brain sections were immediately mounted on glass slides, dried at room temperature and then stored at −20 °C. Sections were used for H&E stainings on a Discovery XT automated stainer (Roche Diagnostics Switzerland/Ventana Medical Systems, Tucson, AZ, USA). The annotation of hippocampal structures was performed manually according to Franklin and Paxinos^28^ using DEFINIENS software on H&E stained brain section scans. Every 10^th^ section of 20 µm thick cuts was annotated resulting in 16 to 18 sections per hippocampus. Volumes were calculated with the following formula: ${\rm{V}}=\frac{{\rm{a}}+{\rm{b}}}{2}\times 200\,\mu m$, whereas a and b are the areas of the annotated hippocampi of the brain slides given by the imaging software Definiens Developer XD 2. Single volumes were added up to the total volume of the hippocampus.

### Human subjects

134 healthy volunteers (57 females, 77 males; age: median 27, interquartile range 24–44 years; body mass index [BMI]: median 25.5, interquartile range 22.8–29.0 kg/m²) with available high-resolution T1 images were genotyped for the single nucleotide polymorphism (SNP) rs2334499:C > T (NC_000011.9:g.1696849 C > T, minor allele frequency 0.45, SNP was in Hardy-Weinberg equilibrium [p = 0.76]). There were no significant differences in age or BMI between genotypes (p > 0.5). For genotyping, DNA was isolated from whole blood using a commercial DNA isolation kit (NucleoSpin, Macherey & Nagel, Düren, Germany). The SNP rs2334499:C > T was genotyped using the Agena MassARRAY^®^ System with iPLEX software (Agena Bioscience GmbH, Hamburg, Germany). All subjects provided informed written consent and the local ethics committee at the University of Tübingen approved the protocol. Human studies were performed in accordance with the relevant guidelines and regulations.

### Voxel based morphometry

High-resolution T1-weighted images (1 × 1 × 1 mm^3^) were acquired at a 3 Tesla Siemens scanner (3 Tesla Tim Trio and PRISMA; Magnetization Prepared Rapid Acquisition Gradient Echo sequence (MPRAGE)). The T1 weighted images were processed and examined using the VBM8 toolbox segmenting structural images into gray matter (GM), white matter (WM) and cerebrospinal fluid (CSF) and using Diffeomorphic Anatomical Registration Through Exponentiated Lie algebra (DARTEL)^29^. DPABI was used to implement VBM8^30^. GM volume was extracted of the whole cerebrum and three hippocampal subregions (Cornu Ammonis (CA) 1–3, CA4-dentate gyrus, and subiculum; separately for left and right regions each) based on the computational atlas by Iglesias, *et al*.^31^ for further statistical analyses.

### RNA isolation and qPCR

RNA of microdissected hippocampal tissues was extracted using the NucleoSpin RNA extraction kit (Macherey-Nagel GmbH + Co. KG, Düren, Germany) according to the manufacturers protocol. RNA was eluted in 40 μl pre-warmed RNase-free water. QuantiTec Reverse Transcription reagents (Qiagen N.V., Hilden, Germany) were used to transcribe 0.6 μg of RNA to cDNA. The following TaqMan Assay probes were used: Dusp8 (Mm01158980_m1) and HPRT (Mm01545399_m1). The delta-delta cycle threshold (Ct) method was used for evaluating gene expression levels. Hypoxanthine phosphoribosyltransferase 1 (HPRT) was used as a reference housekeeping gene. Quantitative PCRs were conducted with a Viia7 cycler (Applied Biosystems/Thermo Fisher Scientific Inc.).

### Statistical analysis

For statistical analyses GraphPad Prism 7.0c, JMP 13 (SAS Institute, Cary, NC) or SPSS (IBM, Armonk, NY, USA) were used. Multiple comparisons were performed by Two-way ANOVA with ad hoc Sidak’s multiple comparison tests. Two-tailed unpaired Student’s t-test were used to compare two groups. Murine hippocampal weights and volumes were further assessed by Analysis of Co-Variance (ANCOVA) and skull length and/or body weight as covariates. Associations of genetic variants with human brain volumes were analyzed by multiple linear regression analyses using additive inheritance model. Human data were log_e_-transformed prior to analyses. P-values ≤ 0.05 were considered as statistically significant. Unless stated otherwise, all results are presented as means ± SEM.

## Supplementary information

Supplementary Info

## Data Availability

The datasets generated during and/or analyzed during the current study are available from the corresponding author on reasonable request.
