# Supplementary material for: Dusp8 affects hippocampal size and behavior in mice and humans
Source: Sci Rep. 2019 Dec 20;9:19483. doi: 10.1038/s41598-019-55527-7 (PMC6925303; doi:10.1038/s41598-019-55527-7)
Supplement: Supplementary file 1 — Supplementary Info [file 41598_2019_55527_MOESM1_ESM.pdf]

Dusp8 affects hippocampal size and behavior in mice and humans

Peter Baumann[1,2,3,4], Sonja C. Schriever[1,2,3], Stephanie Kullmann[3,5,6], Annemarie Zimprich[7,8,9], Annette Feuchtinger[10], Oana Amarie[7], Andreas Peter[3,5,11], Axel Walch[10], Valerie Gailus-Durner[7], Helmut Fuchs[7], Martin Hrabe de Angelis[3,7,12], Wolfgang Wurst[8, 9,13,14], Matthias H. Tschöp[2,3,15], Martin Heni[3,5,6], Sabine M. Hölter[7,8,9], and Paul T. Pfluger*[1,2,3,4]

1: Research Unit Neurobiology of Diabetes, Helmholtz Zentrum München, 85764, Neuherberg, Germany; 2: Institute for Diabetes and Obesity, Helmholtz Zentrum München, 85764, Neuherberg, Germany; 3: German Center for Diabetes Research (DZD), 85764, Neuherberg, Germany; 4: Neurobiology of Diabetes, TUM School of Medicine, Technische Universität München, 80333, Munich, Germany; 5: Institute for Diabetes Research and Metabolic Diseases (IDM) of the Helmholtz Center Munich at the University of Tübingen, 72076, Tübingen, Germany; 6: Department of Internal Medicine IV, University Hospital of Tübingen, 72076, Tübingen, Germany; 7: German Mouse Clinic, Institute of Experimental Genetics, Helmholtz Zentrum München, 85764, Neuherberg, Germany; 8: Institute of Developmental Genetics, Helmholtz Zentrum München, 85764, Neuherberg, Germany; 9 Chair of Developmental Genetics, Technische Universität München-Weihenstephan, c/o Helmholtz Zentrum München, 85764, Neuherberg, Germany; 10: Research Unit Analytical Pathology, Helmholtz Zentrum München, 85764, Neuherberg, Germany; 11: Institute for Clinical Chemistry and Pathobiochemistry, Department for Diagnostic Laboratory Medicine, University Hospital of Tübingen, 72076, Tübingen, Germany; 12: Chair of Experimental Genetics, School of Life Science Weihenstephan, Technische Universität München, 85354 Freising, Germany; 13: German Center for Neurodegenerative Diseases (DZNE) Site Munich, 81377, Munich, Germany; 14: Munich Cluster for Systems Neurology (SyNergy), Ludwig-Maximilians-Universität München, 81377, Munich, Germany; 15: Division of Metabolic Diseases, Technische Universität München, 80333, Munich, Germany

*corresponding author

peter.baumann@helmholtz-muenchen.de; sonja.schriever@helmholtz-muenchen.de; Stephanie.Kullmann@med.uni-tuebingen.de; annemarie.zimprich@helmholtz-muenchen.de; annette.feuchtinger@helmholtz-muenchen.de; oana-veronica.amarie@helmholtz-muenchen.de; Andreas.Peter@med.uni-tuebingen.de; axel.walch@helmholtz-muenchen.de; gailus@helmholtz-muenchen.de; hfuchs@helmholtz-muenchen.de; hrabe@helmholtz-muenchen.de; wurst@helmholtz-muenchen.de; matthias.tschoep@helmholtz-muenchen.de; Martin.Heni@med.uni-tuebingen.de; hoelter@helmholtz-muenchen.de; paul.pfluger@helmholtz-muenchen.de

Supplement

Figures

Suppl. Figure 1

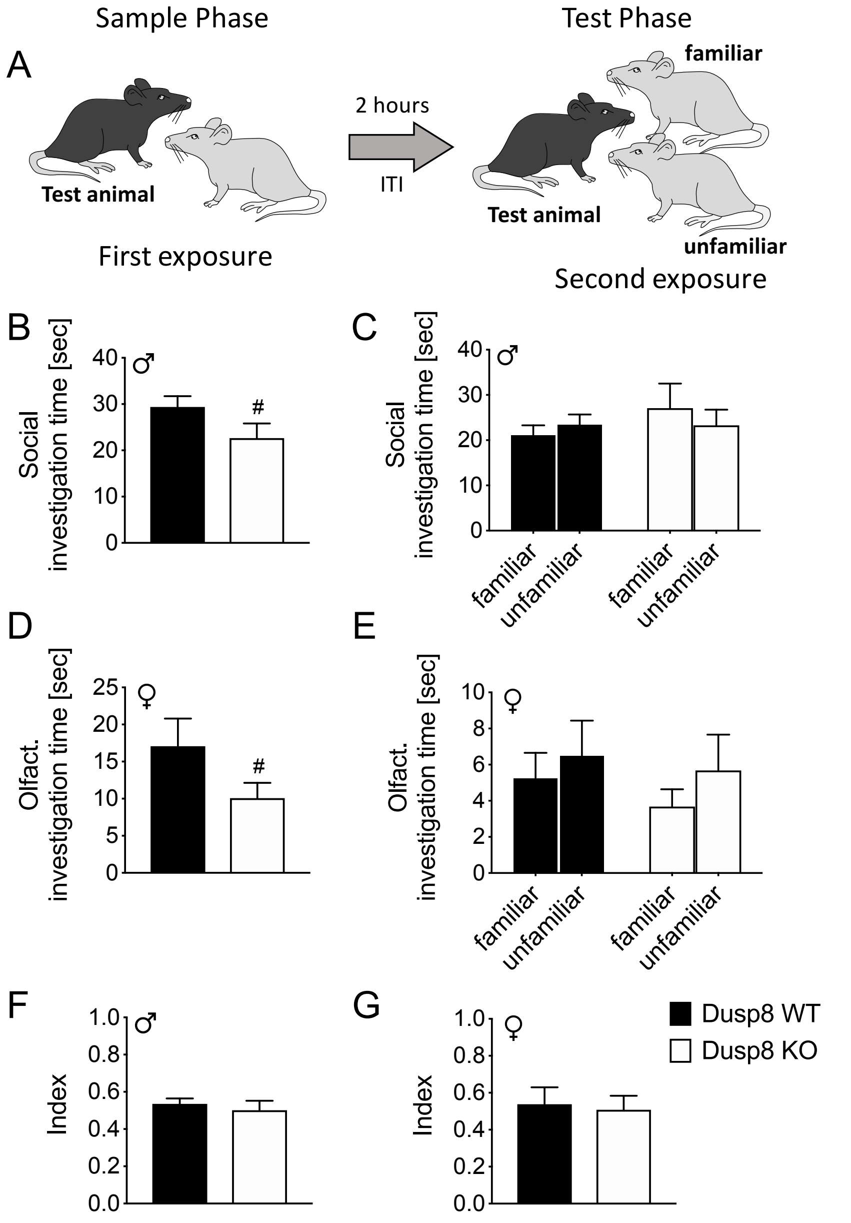

Suppl. Figure 1. Social Discrimination Test in female and male Dusp8 WT and KO mice. (A) Schematic overview of test animals (dark grey) exposed to an unfamiliar ovariectomized female (light grey). Mice were allowed to freely interact for 4 min, followed by a 2-hour inter-trial interval (ITI). Subsequently, the same now familiar mouse and a new unfamiliar ovariectomized female were introduced to the test mouse for 4 min, and the interaction times were recorded. The interaction time of male Dusp8 WT or KO mice with the unfamiliar animals is depicted in the sample phase (B) or the test phase (C). The olfactory interaction times of female Dusp8 WT and KO mice are depicted in the sample (D) and test phase (E). Social investigation times included mounting behavior of male mice in addition to olfactory interactions. (F) shows the social discrimination index of male and (G) female Dusp8 WT and KO mice, respectively, calculated as ratio of interaction time with the unfamiliar mouse and the total interaction time with both mice. Male WT: n=15, male KO: n=14; female WT: n=8, female KO: n=12. Means ± SEM. # $p < 0.1$. Elements of artwork were designed by PB.

Suppl. Figure 2

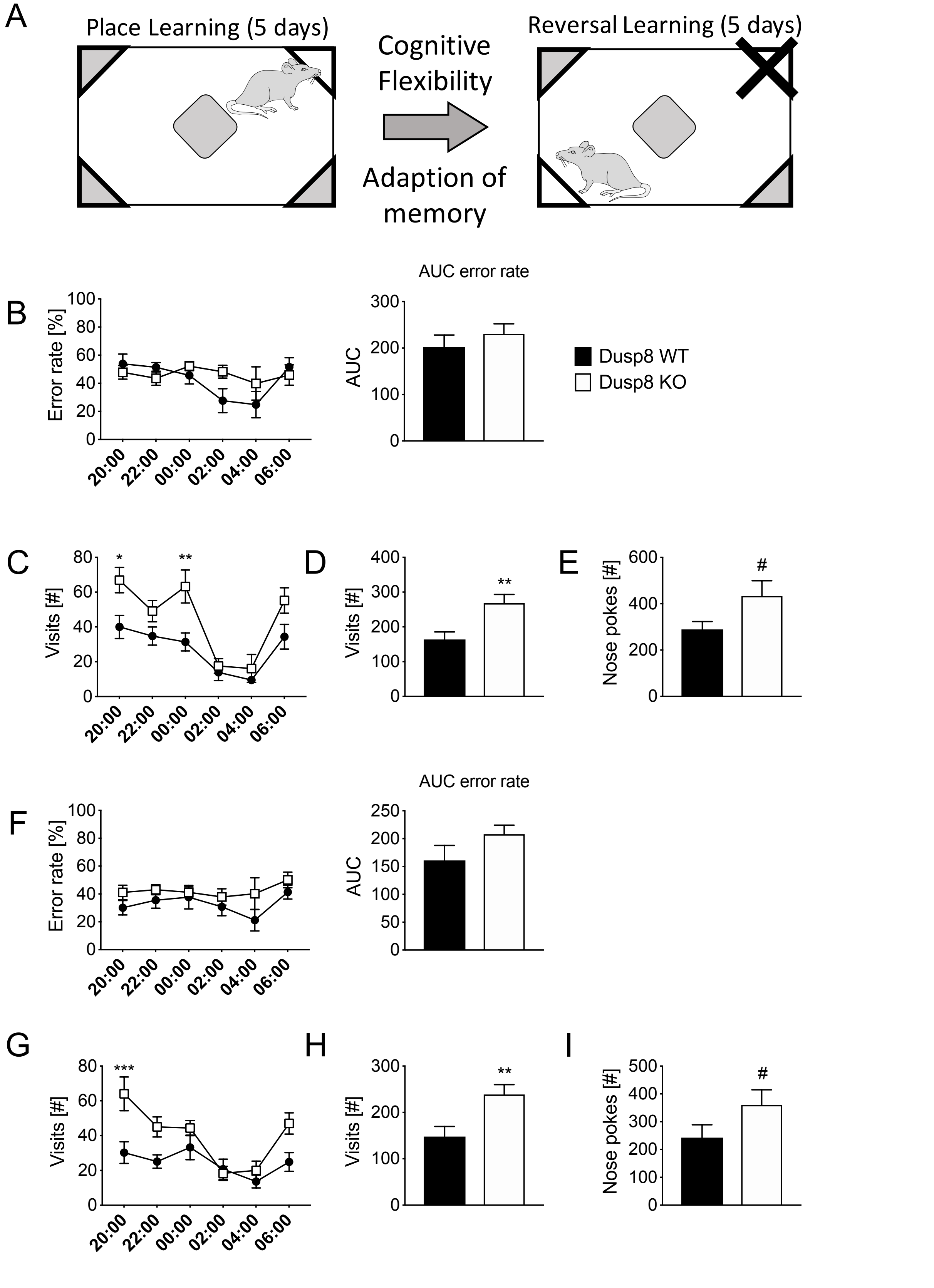

Suppl. Figure 2. Reversal Learning Test of female Dusp8 WT and KO mice group-housed in an automated IntelliCage behavioral monitoring system. (A) Schematic of the reversal learning task. In the initial place learning test, one corner (labelled in white) contains a water bottle with free access after a nose poke, the other three (grey) corners contain bottles with blocked access. To induce reversal learning, the accessible bottle in the (white) corner is switched to the opposing corner, which will now grant access to water after a nose poke. Subsequently, (B) the

number of erroneous nose pokes in the first night phase as measure for the reversal learning performance, and (C-E) the overall activity of the mice in the first night phase, monitored as bi-hourly number of corner visits (C), average number of corner visits (D) and average number of nose pokes (E), were recorded. (F-I) depict the corresponding values from the second night of the reversal learning task. WT: n=8, KO: n=9. Means ± SEM. # p < 0.1; * p < 0.05; ** p < 0.01, *** p < 0.001. Elements of artwork were designed by PB.

Suppl. Figure 3

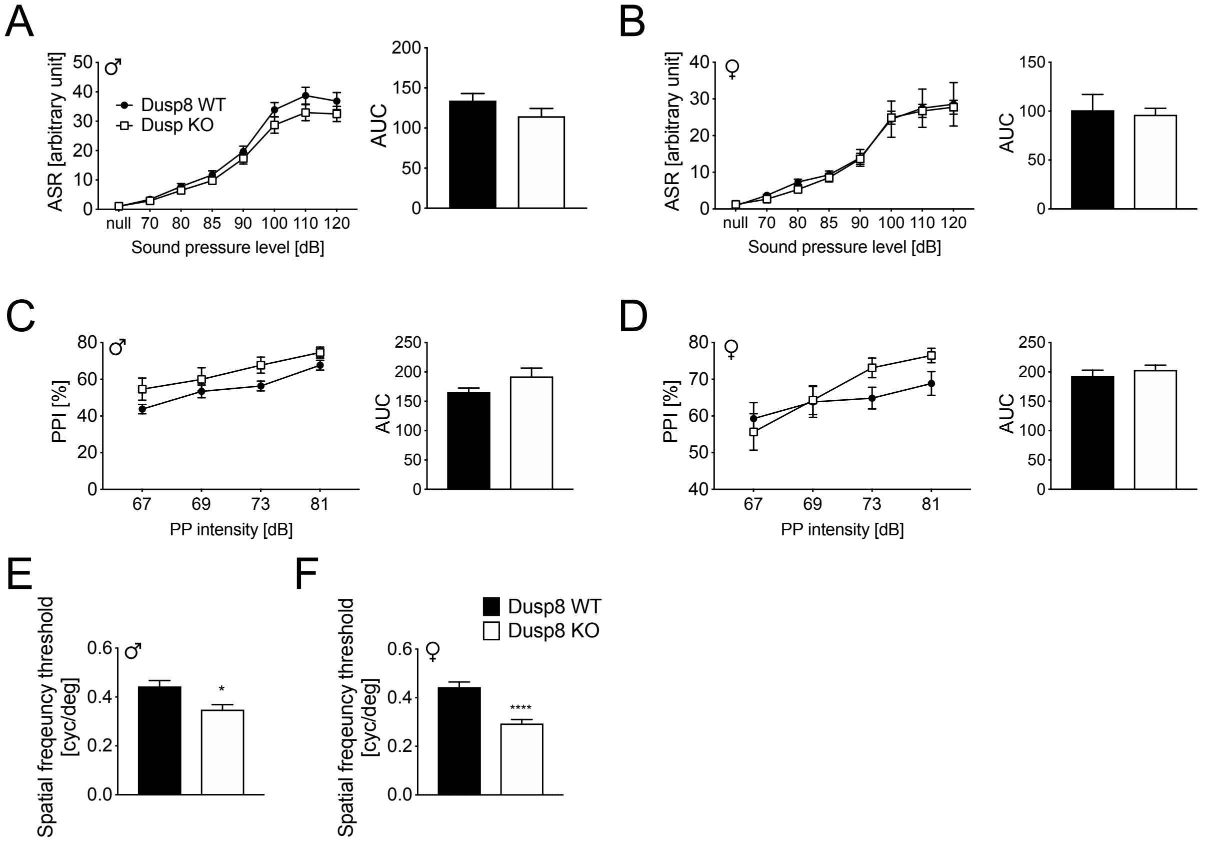

Suppl. Figure 3. Acoustic Startle Response, Prepulse Inhibition and Virtual Drum Test. The ASR was measured at increasing levels of stimuli varying from 70 dB to 120 dB with a background level of 65 dB. Neither male (A) nor female (C) Dusp8 KO mice showed differences in the ASR compared to their WT littermates. The PPI test was conducted with prepulse stimuli ranging from 67 dB to 81 dB with an inter-stimulus interval of 50 ms. PPI was unperturbed in Dusp8 KO mice compared to WT littermates (B,D). Male WT: n=15, male KO: n=15; female WT: n=8, female KO: n=12. Visual acuity in the virtual drum test in male (E) and female (F) Dusp8 KO mice compared to Dusp8 WT littermates. Male WT: n=7, male KO: n=7; female WT: n=7, female KO: n=8. Means ± SEM. *** p < 0.001.

Suppl. Figure 4

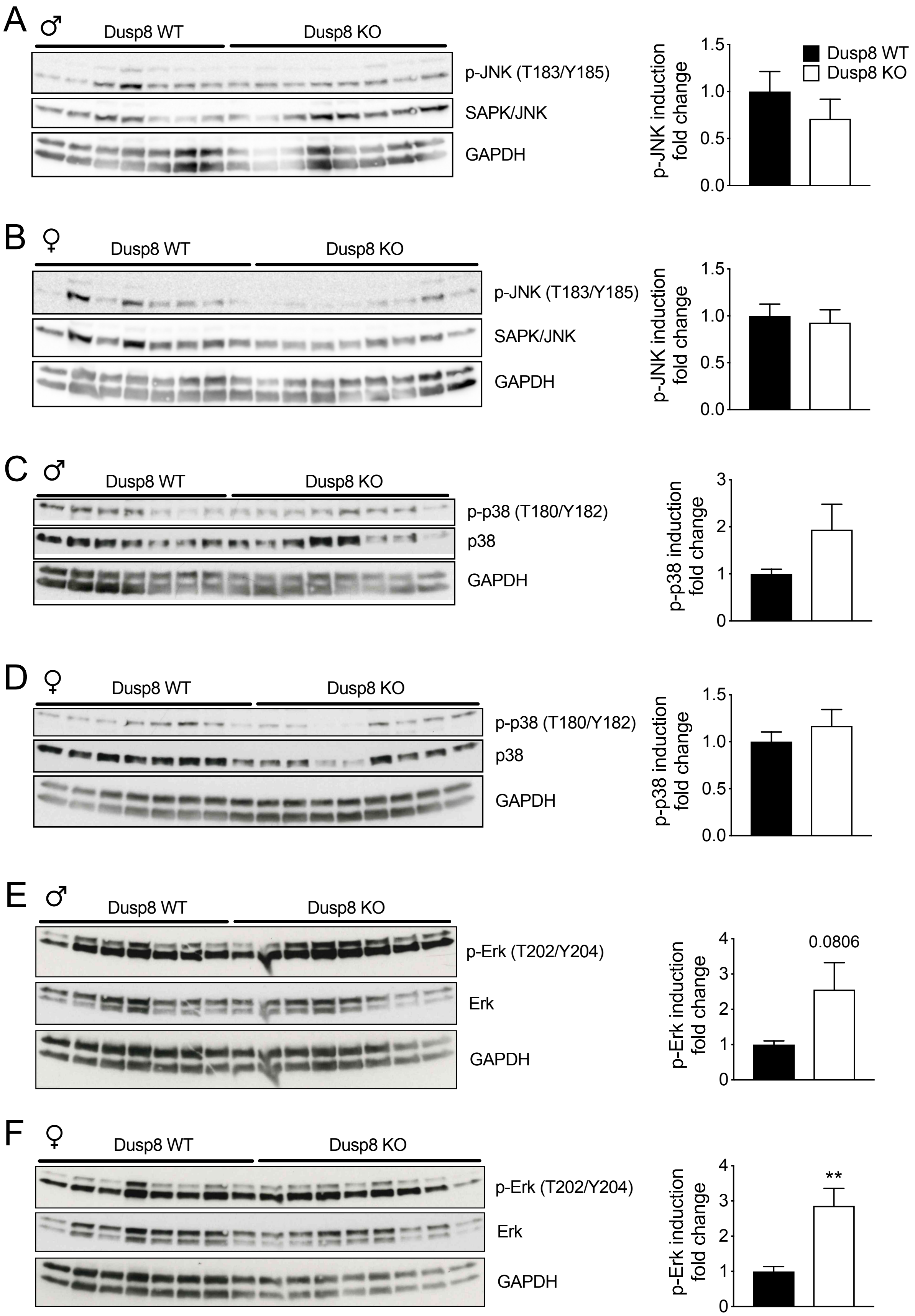

Suppl. Figure 4. MAPK activity in the hippocampus. Densitometric analysis for phosphorylated and non-phosphorylated MAPKs. JNK-signaling in male (A) and female (B) Dusp8 WT and Dusp8 KO mice. Activity of p38-

signaling pathway in male (C) and female (D) Dusp8 WT and Duso8 KO mice. Western blot analysis of Erk-signaling in the Hippocampus of male (E) and female (F) Dusp8 WT and Dusp8 KO mice. Fold change of phosphorylation relative to respective total protein and GAPDH. 30 µg of protein loaded per sample. Original blots are shown in suppl. figure 6-11. Male WT: n=7, male KO: n=8; female WT: n=8, female KO: n=8. Means ± SEM.

Suppl. Figure 5

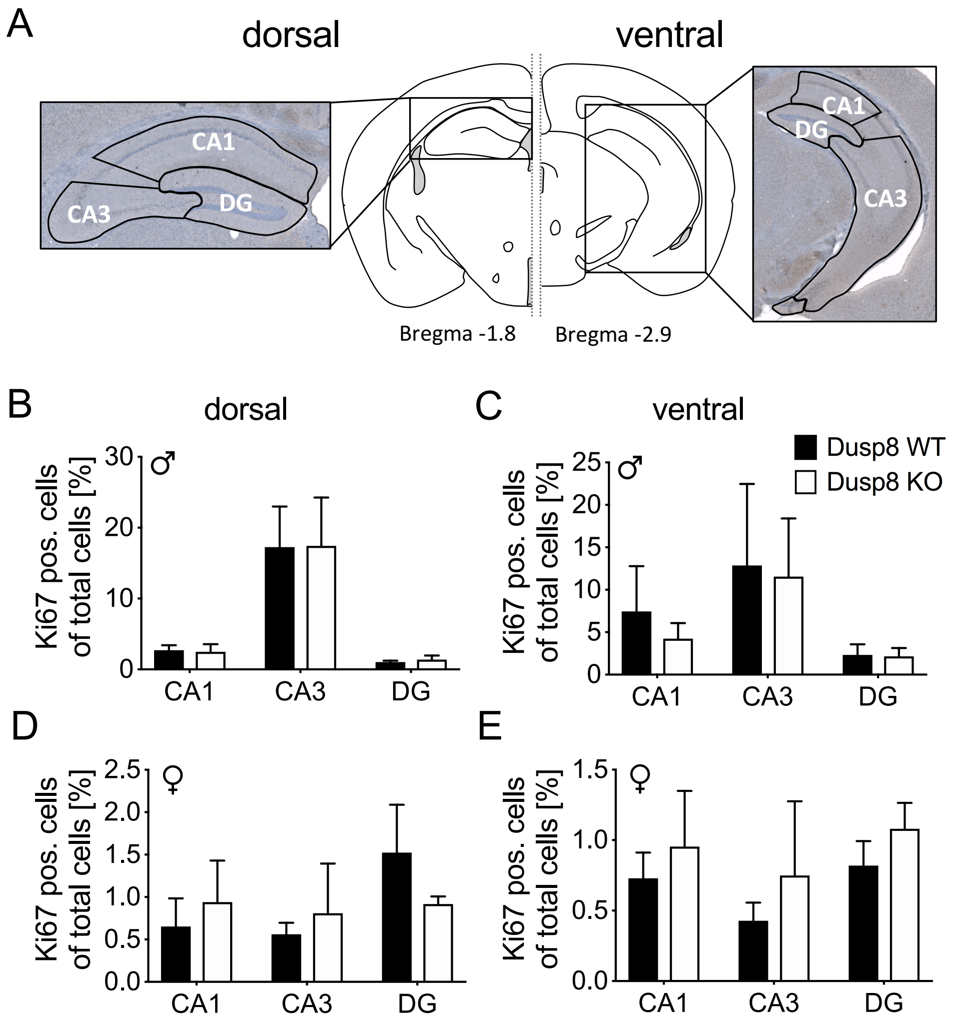

Suppl. Figure 5. Neuronal activity in hippocampal CA1, CA3 and DG regions. Respective regions were manually assigned (A). Immunohistochemical detection of Ki67-positive cells in in the dorsal (B,D) and ventral (C,E) hippocampus of male (B,C) and female (D,E) Dusp8 WT and KO mice. Male WT: n=4, male KO: n=5; female WT: n=3, female KO: n=4-6. Means ± SEM. Elements of artwork were designed by PB.

Suppl.Figure 6

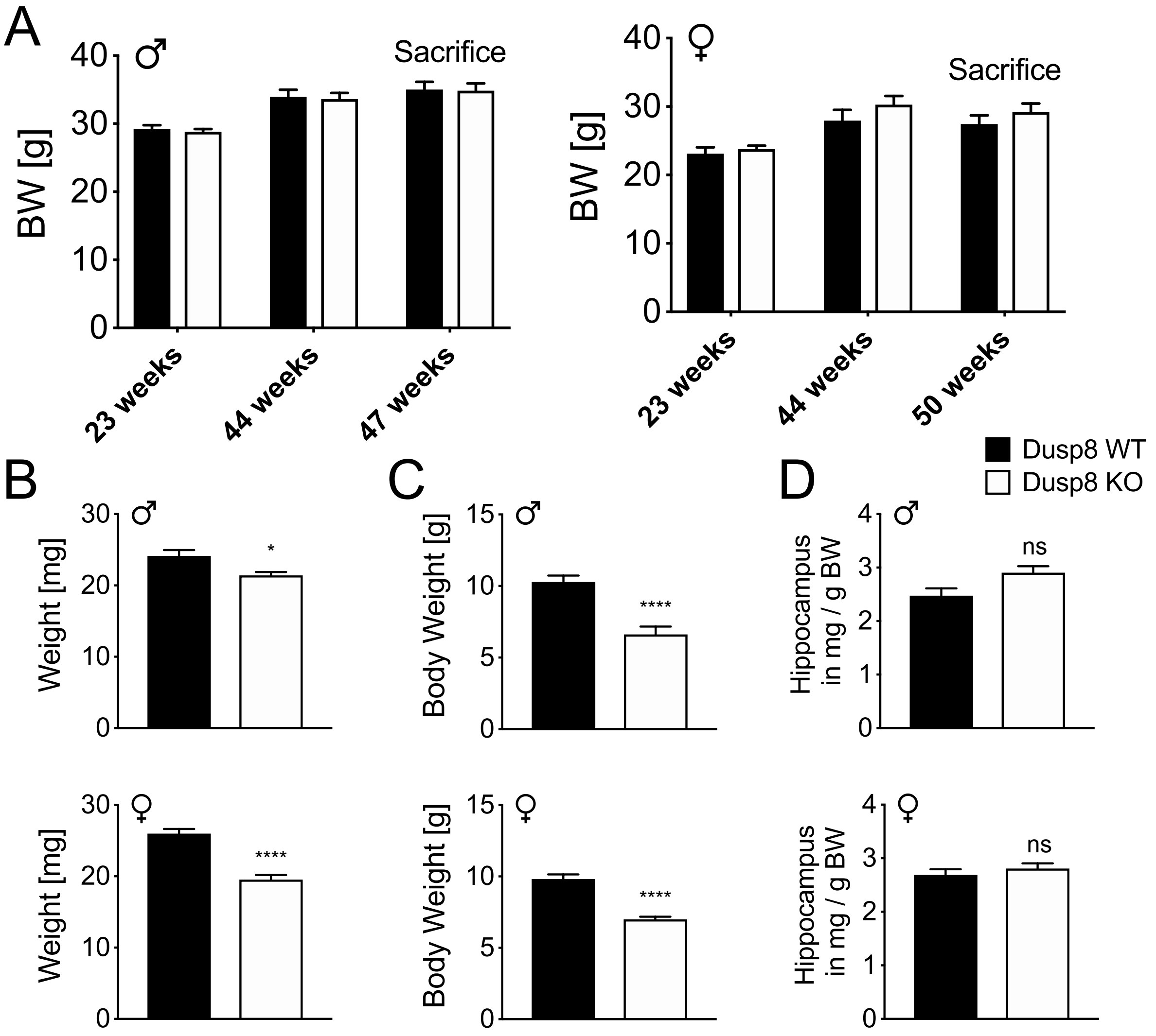

Suppl. Fig. 6. Hippocampus and body weights of chow-fed male and female Dusp8 KO and Dusp8 WT mice. (A) Body weights of Dusp8 KO and Dusp8 WT mice at 23, 44 and at the day of the sacrifice. Male WT: n=14-15, male KO: n=14; female WT: n=8, female KO: n=12. (B) Microdissected hippocampus weights of young mice at 21 days of age (d21). (C) Respective body weight of d21 mice. (D) Normalization of the hippocampus weight to body weight of d21 mice. Male WT: n=16, male KO: n=8; female WT: n=15, female KO: n=10. Means ± SEM. * $p < 0.05$, *** $p < 0.001$, **** $p < 0.0001$, ns not significant.

Suppl. Figure 7

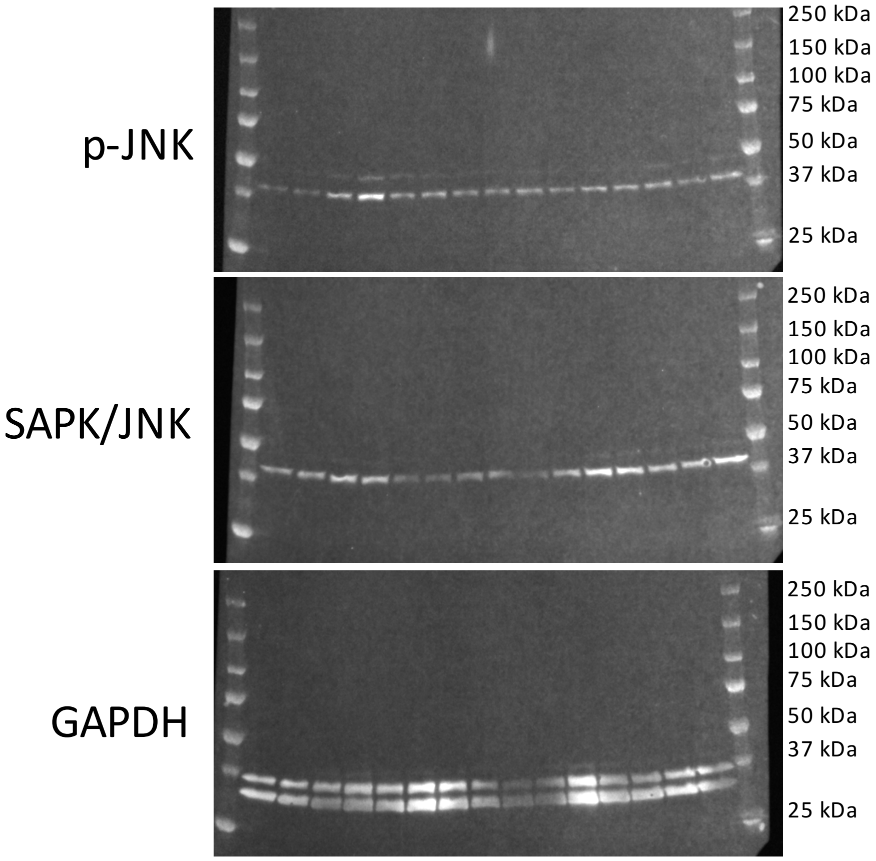

Suppl. Figure 7. Original non-cropped blots of Suppl. Figure 4A. Membrane was stripped with stripping buffer between the respective antibody incubation times and detection.

Suppl. Figure 8

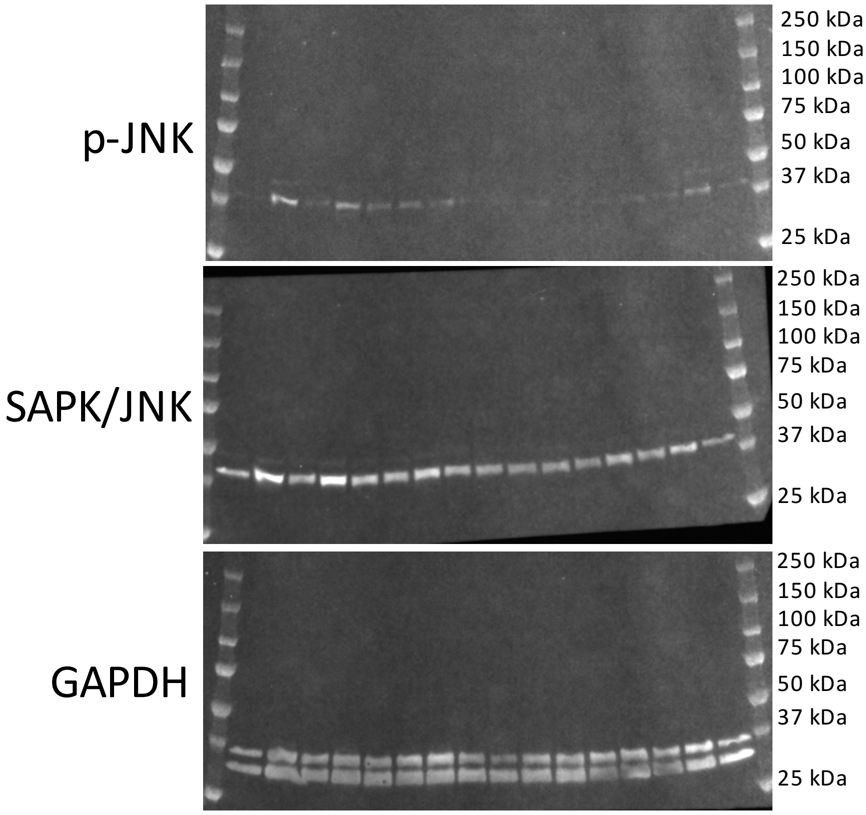

Suppl. Figure 8. Original non-cropped blots of Suppl. Figure 4B. Membrane was stripped with stripping buffer between the respective antibody incubation times and detection.

Suppl. Figure 9

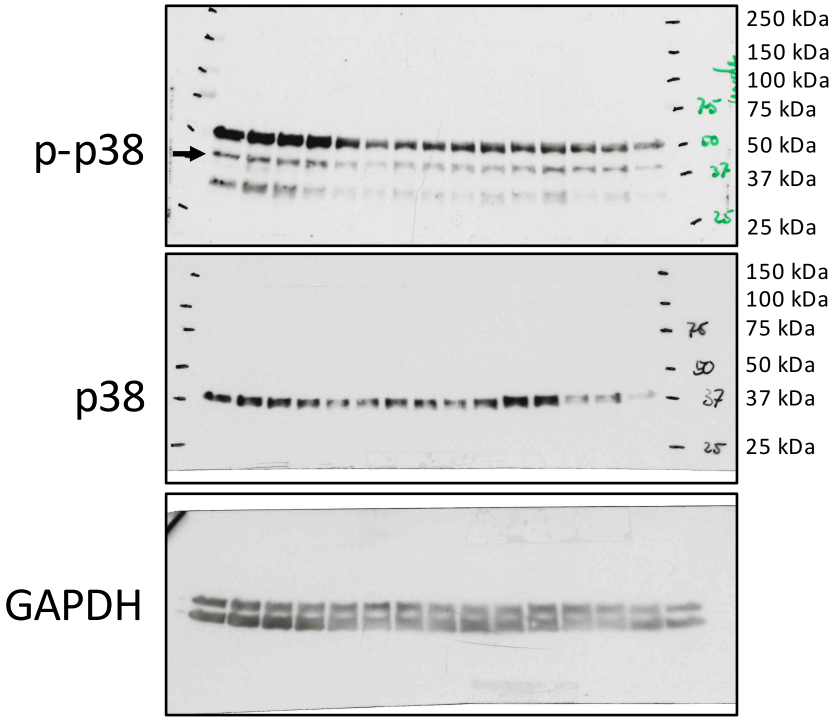

Suppl. Figure 9. Original non-cropped blots of Suppl. Figure 4C. Membrane was stripped with stripping buffer between the respective antibody incubation times and detection. Arrow indicates specific band for p-p38.

Suppl. Figure 10

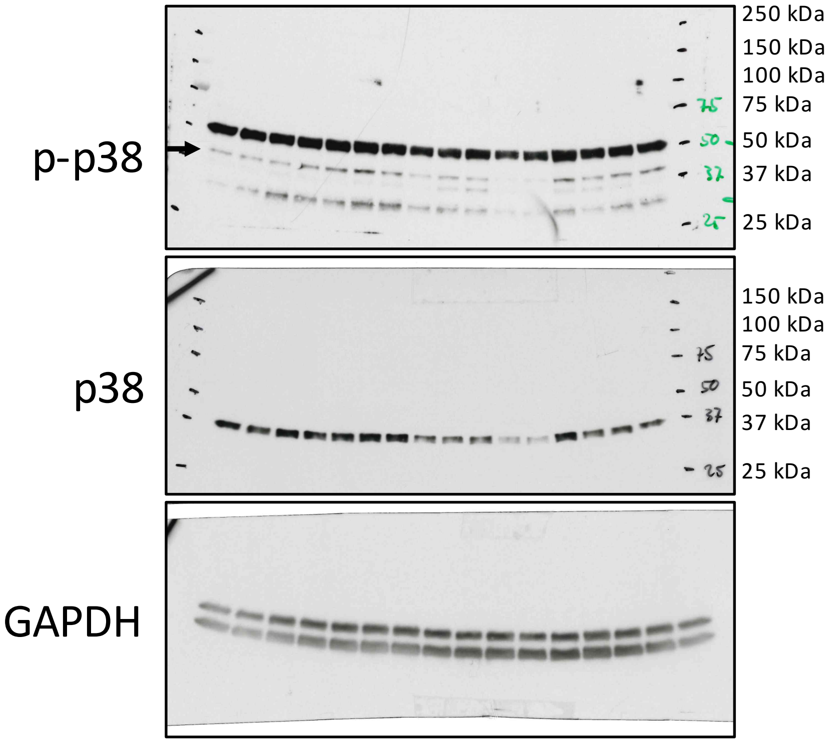

Suppl. Figure 10. Original non-cropped blots of Suppl. Figure 4D. Membrane was stripped with stripping buffer between the respective antibody incubation times and detection. Arrow indicates specific band for p-p38.

Suppl. Figure 11

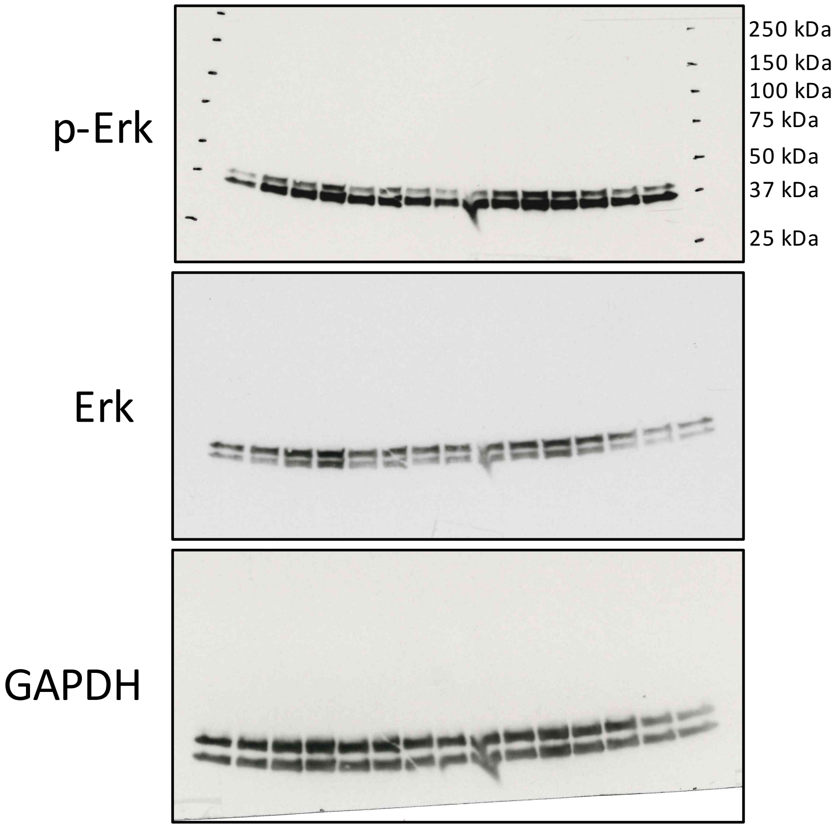

Suppl. Figure 11. Original non-cropped blots of Suppl. Figure 4E. Membrane was stripped with stripping buffer between the respective antibody incubation times and detection.

Suppl. Figure 12

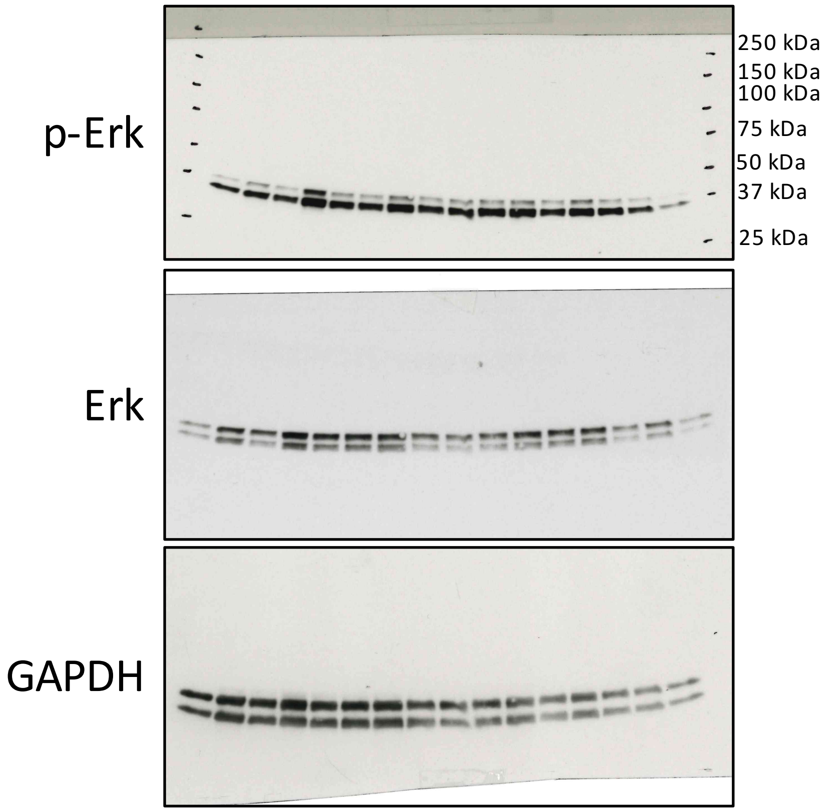

Suppl. Figure 12. Original non-cropped blots of Suppl. Figure 4F. Membrane was stripped with stripping buffer between the respective antibody incubation times and detection.

Methods

Social discrimination

Social discrimination tests were performed and analyzed as described[1] and conducted between 08.00 am – 02.00 pm. Briefly, for the sample phase an ovariectomized female stimulus mouse was exposed to the test mouse in a neutral cage for 4 min. After a 2-h inter-trial-interval both the familiar and a new unfamiliar stimulus mouse were placed into a neutral cage to the test mouse for another 4 min. Interactions of the test mouse with the stimulus mice were tracked manually with a handheld computer PSION Teklogix Workabout Pro (Noldus Pocket Observer Version 2.1.23.f) during both sample and test phases.

Reversal Learning Task

Immediately after the Place Learning task, mice were subjected to the Reversal Learning task. Briefly, each mouse was assigned to the opposite corner of the cage for access to the drinking bottles after a nose poke, and nose pokes and corner visits were monitored for 5 d. Mice were not disturbed or handled during testing days. Both tests were performed according to Holter, et al. [1].

Acoustic Startle Response and Prepulse Inhibition

Acoustic startle response (ASR) and Prepulse Inhibition (PPI) were assessed to investigate sensorimotor gating. Mice were placed in an animal holder on a load cell platform within a dark sound attenuating chamber. The load cell platform was used to measure physical movement of the mice triggered by acoustic stimuli. A background noise of 65 dB was set for the whole session. Measurements for ASR and PPI were arranged in a pseudo-random order and organized in 10 blocks, each presented 10 times after an initial stimulus-free acclimation period of 5 min. For ASR acoustic stimulus levels of 70, 80, 85, 90, 100, 110, and 120 dB were presented. PPI was assessed for an acoustic stimulus level of 110 dB with an inter-stimulus interval of 50 ms. Inhibition was measured for prepulse levels of 67, 69, 73, and 81 dB. Each testing block was started with a presentation of the startle stimulus alone (110 dB) five times.

Virtual Drum Test

The Optomotor System (Striatech, Tübingen, Germany) was utilized to record mouse visual acuity via recording of optokinetic responses (OKR)[2]. The moving grid pattern

invokes slow eye and head movements in the direction of rotation. Monitoring the head position of a freely moving animal inside the system with a camera, the virtual drum test allows for recognition of specific spatial frequencies (cycles/degree) that are indicated by a tracking movement of the rodent's head. The system automatically evaluates the reaction to the change, which increases or decreases the stimulus frequency until a spatial frequency threshold is attained. Spatial frequency thresholds were recorded at 100% contrast and a constant drift speed at 12.0 d/s.

Protein extraction and western blotting

Ten days after the last test day whole hippocampus microdissections were performed from fresh brain tissue. Hippocampus tissue was lysed in RIPA buffer (Thermo Fisher Scientific Inc., Waltham, MA, USA) containing a phosphatase and protease inhibitor cocktail Thermo Fisher Scientific) and 1 mM phenyl-methane-sulfonylfluorid (PMSF). Samples were then centrifuged (12,000x g, 7 min) and supernatants were collected. Samples were equally diluted in lysis buffer and 4x NuPage buffer (Thermo Fisher Scientific) + 50 µM DTT. After denaturation samples were loaded and separated on a 4-20% gradient Criterion™ TGX™ Precast Gels (Biorad, Munich, Germany). Proteins were transferred to a PVDF membrane and blocked in Tris-buffered-saline with 0.05% Tween 20 (TBS-T) containing 5% BSA for 1 h. Proteins were detected by the following primary antibodies diluted in blocking buffer: anti-phospho-SAPK/JNK (polyclonal rabbit, 1:1000, Cat #9251, Cell Signaling Technology, Danvers, MA, USA), anti-JNK1 (monoclonal mouse, 1:1000, Cat #3708, Cell Signaling Technology), anti-phospho-Erk (polyclonal rabbit, 1:1000, Cat #9101, Cell Signaling Technology), anti-Erk (polyclonal rabbit, 1:1000, Cat #9102, Cell Signaling Technology) anti-phospho-p38 polyclonal rabbit, 1:1000, Cat #4511, Cell Signaling Technology), anti-p38 (polyclonal rabbit, 1:1000, Cat #9212, Cell Signaling Technology), and anti-GAPDH (monoclonal mouse, 1:10,000, Cat sc166545; Santa Cruz Biotechnology, Dallas, TX, USA). Secondary antibodies anti-rabbit HRP (goat anti-rabbit IgG-HRP, Cat sc-2004, Santa Cruz Biotechnology), and anti-mouse HRP (mIgGk BP-HRP, Cat sc-516102, Santa Cruz Biotechnology) were diluted 1:10 000. ECL Clarity (Biorad) was used for detection of HRP-induced chemiluminescence with the Fluorescence-chemiluminescence system LI-COR Odyssey Fc (LI-COR GmbH, Bad Homburg, Germany) and imaging software iS Version 5.2 or Amersham Hyperfilm ECL (#28906836, GE Healthcare Limited, Little Chalfont, UK). After every detection, membranes were incubated for 30 min with

Restore PLUS Western Blot stripping buffer (#46430, Thermo Fisher Scientific). Densitometric analyses were performed with ImageJ (2.0.0-rc-68/1.52f).

Immunohistochemistry

Mouse brains were harvested and processed according to the described method in 4.3 Hippocampus volume calculation. The following antibodies were employed for immunohistochemistry: rabbit anti-Ki67 (1:1000, ab15580, Abcam, Cambridge, UK) or rabbit anti-Casp3 (1:100, Cat #9664, Cell Signaling Technology) as primary antibodies or anti-rabbit (1:750, BA 1000, Vector Laboratories, Burlingame, USA) as secondary antibody. Detections were carried out with a Discovery® DAB Map Kit (Roche Diagnostics, Switzerland / Ventana Medical Systems).

Image-based automated cell counting

The stained tissue sections were scanned at 20× objective magnification using an AxioScan.Z1 digital slide scanner (Zeiss, Oberkochen, Germany). Images were evaluated using the commercially available image analysis software Definiens Developer XD 2 (Definiens AG, Munich, Germany) following a previously published procedure[3]. Regions of interest, e.g. specific brain areas, were annotated manually. The calculated parameters within these defined regions were the ratio of positively immunostained cells per total cell.

References

1 Holter, S. M. *et al.* Assessing Cognition in Mice. *Current protocols in mouse biology* **5**, 331-358, doi:10.1002/9780470942390.mo150068 (2015).

2 Prusky, G. T., Alam, N. M., Beekman, S. & Douglas, R. M. Rapid quantification of adult and developing mouse spatial vision using a virtual optomotor system. *Investigative ophthalmology & visual science* **45**, 4611-4616, doi:10.1167/iovs.04-0541 (2004).

3 Feuchtinger, A. *et al.* Image analysis of immunohistochemistry is superior to visual scoring as shown for patient outcome of esophageal adenocarcinoma. *Histochemistry and cell biology* **143**, 1-9, doi:10.1007/s00418-014-1258-2 (2015).
